# Neuroretinitis and chorioretinitis in a cat-scratched young boy: a case report

**DOI:** 10.22336/rjo.2025.17

**Published:** 2025

**Authors:** Cristina-Ariadna Nicula, Adina-Ioana Lăpuște, Ariana-Ioana Lăpușan

**Affiliations:** 1 Department of Maxillo-Facial Surgery and Radiology, “Iuliu Hațieganu” University of Medicine and Pharmacy Cluj-Napoca, Romania; 2 County Emergency Hospital, Eye Clinic Cluj-Napoca, Romania

**Keywords:** neuroretinitis, chorioretinitis, cat-scratched, BVA = best visual acuity, ARM = autorefractometry, RE = right eye, LE = left eye, OCT = optical coherence tomography, VF = visual field, CS = corticosteroids, HHC = hydrocortisone hemisuccinate, AB = antibiotics

## Abstract

Neuroretinitis is an inflammatory type of optic nerve damage evidenced by the appearance of papillary edema. It also involves inflammation of the retinal layers, as evidenced by the thickening of these layers and the presence of intra- and subretinal fluid. Chorioretinitis is a condition in which inflammation of the posterior component of the uvea, the choroid, leads to further damage to the retina, causing it to become inflamed.

The most common causes of neuroretinitis and chorioretinitis in the pediatric population are represented by infectious etiologies. Most cases of neuroretinitis in children are caused by cat scratch disease, which is typically attributed to the bacterium Borrelia burgdorferi.

We present the case of a 10-year-old child who presented to our service complaining of a sudden decrease in vision and the appearance of a central scotoma two days before he was referred to our service. Our first diagnosis was of optic neuritis, based on the presence of objective papillary edema on fundus examination. Subsequently, the appearance of chorioretinal foci completely changed the diagnosis and treatment in this case.

## Introduction

Neuroretinitis is a condition characterized by inflammation of the optic nerve, often accompanied by retinal changes, particularly in the macular region of the retina. The following triad characterizes it: decreased visual acuity, papillary edema, and exudates that cause the appearance of the macular star known as “stellate maculopathy” [1].

Often, the etiology of neuroretinitis remains uncertain. Its possible causes include infectious, non-infectious, and idiopathic etiology. Infectious causes include bacterial (Bartonella henselae, Tuberculosis spp., and Salmonella), viral (Epstein-Barr virus, Cytomegalovirus, and Herpes simplex virus), protozoal (Toxoplasma gondii), and other parasitic infections. Non-infectious causes include malignancies, diabetes mellitus, and systemic or vascular diseases [2].

Chorioretinitis is the inflammation of the choroid, which is the component that provides nourishment to the retina, and whose inflammation, once developed, affects the overlying retina and causes decreased visual acuity. Toxoplasmic chorioretinitis appears to be the most common form of chorioretinitis worldwide. Studies indicate that it is responsible for approximately 15% of blindness cases in the USA [3].

Toxoplasmosis is an infection with an intracellular parasite, Toxoplasma gondii, which can be acquired through ingestion of inadequately thermally prepared food or contact with contaminated fruits and vegetables from cat feces. The infection can also be contracted during intrauterine life, following the transfer of parasite antigens from the mother to the fetus. Most infections may go unnoticed or can cause flu-like symptoms [4]. Ocular toxoplasmosis generally involves the choroid and retina. Still, it may also present in atypical forms, such as optic nerve damage, the absence of chorioretinal scarring, the development of retinal neovascularization, and the lack of vitritis [5].

## Case-report

**Fig. 1:**
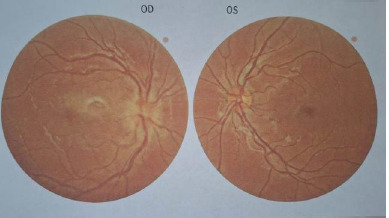
RE papilledema

A 10-year-old boy presented to our clinic on April 27th, 2024, complaining of decreased vision and the appearance of a central scotoma in his right eye (RE) about 2 days before. Before the presentation, he was consulted in another service where the diagnosis of optic neuritis in the RE was established, and he was subsequently redirected to our service (**Fig. 1**).

Ophthalmologic examination on admission revealed: RE BVA = 0.2 without correction, left eye (LE) BVA = 1 (at the Snellen charts). The autorefractometry (ARM) results showed a right eye (RE) of +0.75/-0.50x173° and a left eye (LE) of +0.50/-0.50x3°. For both eyes, the anterior pole was in normal relations at the slit lamp examination. The fundoscopy of the RE revealed an optic disk with a faded contour and a macular area with an intense foveolar reflex without other pathologic aspects (**Fig. 1**). The fundus of the LE was normal.

In these conditions, we established a diagnosis of anterior optic neuritis in the right eye and small hyperopia in both eyes, and we decided to hospitalize the patient in the ophthalmological clinic and initiate treatment.

**Fig. 2:**
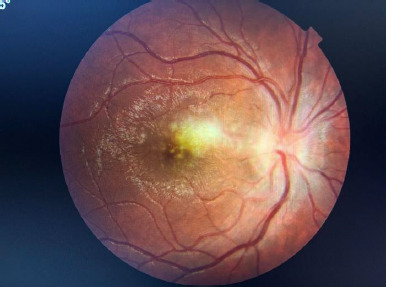
Fundoscopy of RE shows a supero-nasal macular chorioretinal lesion and stellate perimacular exudates

Two days after hospitalization, the funduscopic examination of the RE revealed the appearance of a nasal supero-macular chorioretinal focus, 1 PD size, white-yellowish color, poorly demarcated, stellate dry perimacular exudates (**Fig. 2**). In this case, the diagnosis of RE was established as acute neuroretinitis, acute focal chorioretinitis.

**Fig. 3:**
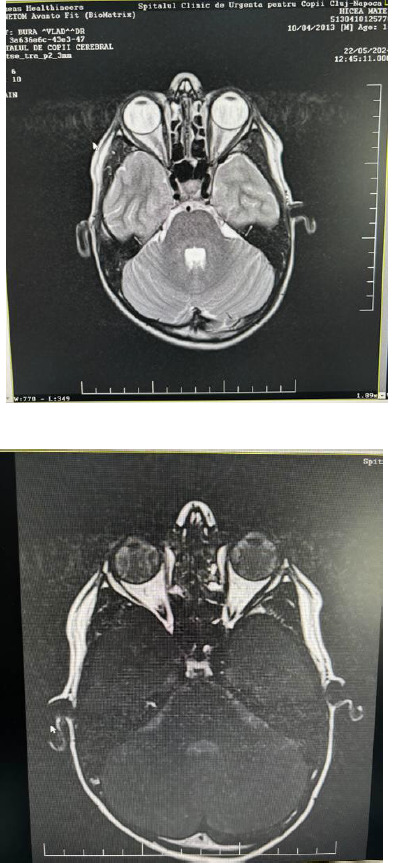
The nuclear magnetic resonance images - normal limits

We further performed laboratory tests to determine the etiology and for differential diagnosis. We analyzed the infectious etiologies, with negative results for Borellia burgdorferi, Bartonella haensele, Herpes simplex virus, and Cytomegalovirus, but with positive IgG antibodies against Toxoplasma gondii. All other tests (hemoleukogram, pulmonary X-ray, dental examination, rheumatologic examination, and cranial nuclear magnetic resonance - **Fig. 3**) were within normal limits.

**Fig. 4:**
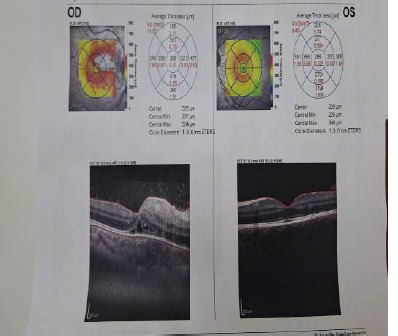
The Macular OCT examination with hyperreflectivity of the retinal layers

Macular optical coherence tomography (OCT) examination revealed the hyperreflectivity of the retinal layers and a few hyperreflective formations localized above the retinal layers at the level of the active areas (**Fig. 4**).

Diagnosis: RE: Acute neuroretinitis. Acute focal chorioretinitis, which was most likely toxoplasmotic.

In this situation, we decided to start steroids treatment with 300 mg/day HHC together with AB treatment with Ceftriaxone 1 g/day and to perform an infectious diseases consultation after which it was decided to start therapy with Clindamycin 30 mg/kg/day, 7 days, and liver protection: Albendazole 400 mg 2x1cpr/day, 5 days, Lagosa 2x1cpr/day, 7 days, Silimarin 3x1/day, 7 days. Seven days after the treatment, the BVA in the RE was 0.3, and a fundoscopy of the RE revealed papillary edema in resorption and a small chorioretinal focus.

Considering the infectious context and the favorable response to treatment, the final diagnosis remained RE: acute neuroretinitis, toxoplasmic acute focal chorioretinitis, acquired toxoplasmosis, BE: Small hyperopia. The patient continued to receive systemic treatment with Prednisone 1 mg/kg body weight, a tapered dose, and gastric protection (Omeprazole 20 mg, one tablet per day).

**Fig. 5:**
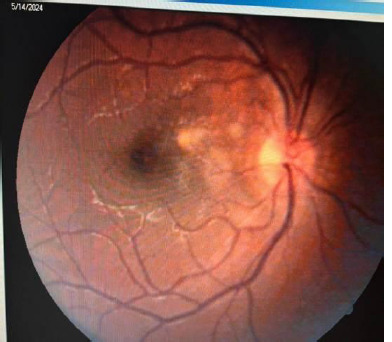
Optic disc without edema, small supero-temporal chorioretinal focus, clear macular area, with discrete foveolar reflex

After one month, the BVA of RE was 100%, and fundoscopy of RE revealed a well-demarcated optic disk without edema, along with a resorbing supero-macular chorioretinal focus (**Fig. 5**).

When a patient has such manifestations, the most crucial question is whether the etiology is infectious or non-infectious.

1. The infectious etiology

Toxoplasmosis infection is the most common cause of posterior uveitis worldwide, affecting 20%-60% of individuals. It presents as necrotizing retinitis with adjacent vitritis, known as “a headlight in the fog”. A pigmented retinochoroidal scar may be present near the lesion, and occasionally, retinal arterial or venous vasculitis or optic nerve edema may occur [6].

Retinitis from “cat scratch disease” is transmitted by biting or scratching a healthy cat, affecting the eyes in 5-10% of cases. It most commonly appears as neuroretinitis, characterized by edema of the optic disk with star-shaped macular exudates [7].

Ocular involvement from Borrelia burgdorferi infection is most commonly manifested by anterior pole involvement, typically presenting as anterior or intermediate uveitis. A distinctive change would be oculomotor nerve palsy, which results from nerve damage from neuroborreliosis [8].

2. Non-infectious etiology

Cases of ocular sarcoidosis range from 12% to 76%, with ocular involvement being the first manifestation in 30-40% of cases. It most commonly causes anterior uveitis, and the most common posterior pole manifestations are those of periphlebitis (extensive perivascular exudation – “candle wax drippings”) and vitritis (inflammatory cells in “snowball” or linear “string of pearls”) and characteristic chorioretinal nodular granulomas [9].

## Discussions

Toxoplasmotic chorioretinitis often presents as a whitish, fluffy area with adjacent retinal necrosis, which causes chorioretinal scarring. Atypical presentations tend to occur in immunocompromised patients, including multifocal chorioretinal lesions, extensive lesions, bilateral involvement, or damage to the optic nerve [10]. In the case of our patient, we can mention an atypical presentation characterized by initial optic nerve damage followed by the appearance of a chorioretinal focus after a few days, which made it difficult to establish a diagnosis and delayed the therapeutic decision.

Even if the onset was atypical, the laboratory tests pointed the diagnosis to toxoplasmosis disease. Immunologic markers can be harmful, especially in immunocompromised individuals, but the most critical factors in this case are the clinical presentation and the patient’s medical history. Currently, PCR can be performed in aqueous humor or in vitro, but it is a laborious procedure, especially in children [10].

The most common cause of neuroretinitis of infectious etiology is neuroretinitis from cat scratch disease. Nausea and fever are common accompanying symptoms. The retinal involvement is characteristic of macular exudates, which on fundoscopy appear as a “macular star” [11]. Thus, it can be stated that our patient could have had an incipient diagnosis of neuroretinitis due to cat scratch disease, but this was ruled out after Bartonella antibodies were performed.

Intraocular inflammation in patients with ocular toxoplasmosis can range from mild to severe, extensive lesions, being responsible for causing more severe vitritis, which leads to the classic “headlight in the fog” sign.

In our case, the absence of vitritis was another clinical impediment that delayed the diagnosis. Recent studies suggest that the absence of vitritis in these patients is most likely due to low immunity. However, even without vitritis, the clinical appearance should not exclude toxoplasmotic disease [12,13].

## Conclusions

Ocular toxoplasmosis is a health problem that can occur at any age and whose management is critical. Early diagnosis and correct treatment can restore patients’ vision, even in the case of severe retinal damage.

Atypical presentations should be considered in any patient with positive Toxoplasma gondii serology.

Patients must be informed about the possibility of relapses, even in the contralateral eye, so that the ophthalmologist can address any change in visual acuity (VA).
